# Use of the STRYD^®^ Power Meter and Its Metrics in Trained Runners: A Systematic Review

**DOI:** 10.3390/sports14080364

**Published:** 2026-08-21

**Authors:** Eugenio Barrios García-Miguel, Carmen Repullo, Teresa Urbina-Gómez, José María González-Ravé

**Affiliations:** 1 Facultad de Ciencias Biomédicas y de la Salud, Universidad Alfonso X el Sabio (UAX), Villanueva de la Cañada, 28691 Madrid, Spain; crepuber@uax.es (C.R.); murbigom@uax.es (T.U.-G.); 2 Sport Training Laboratory, Faculty of Sport Sciences, University of Castilla-La Mancha, 45071 Toledo, Spain; josemaria.gonzalez@uclm.es

**Keywords:** biomechanics, cadence, ground contact time, running power, power output

## Abstract

The STRYD^®^ power meter has been used by runners and running coaches in recent years. The data it provides may offer useful information for training monitoring and performance assessment. Accordingly, this systematic review identifies and analyzes the use of the main parameters provided by the STRYD^®^ power meter in the scientific literature. The PRISMA guidelines were used to identify relevant studies. The search was conducted in the electronic databases PubMed, Scopus and Google Scholar. In total, 142 studies were identified. After removing duplicates and assessing the eligibility of the abstracts, 29 studies were included in the systematic review. Eight of the main metrics provided by STRYD^®^ were analyzed. The total number of times each metric appeared varied widely, as did the number of metrics analyzed in each study. Power output was identified as the most frequently used metric in scientific studies and as the primary variable in the primary objectives sought. Ground contact time and cadence were also used by a high percentage of users, far exceeding the rest: vertical oscillation, flight time, stride length, form power, and stiffness. The study provides coaches and technical staff with guidelines on which variables to use when analyzing runners using STRYD^®^.

## 1. Introduction

Power meters have been used in cycling since the 1980s, when the first portable power meters became commercially available [1]. Today, all professional and amateur cyclists use this technology in their training and competitions. However, the use of portable power meters in running is relatively recent. It was 2014 when the company STRYD^®^ introduced this technology to the international market for general use [2].

Biomechanical analysis in running has evolved significantly in recent years, driven by the development of new technologies that allow for more precise measurement of various kinematic and spatiotemporal parameters. Among these technologies, running power meters have gained prominence due to their ability to estimate the mechanical power generated by runners in real time [3]. These devices provide information that may assist coaches and athletes in adjusting running technique and training load, potentially contributing to improved efficiency [4].

Power measurement in endurance runners has a long history. By 2006, algorithms had already been developed to determine this variable. These algorithms formed the basis for those used in the earliest versions. Subsequent updates incorporated new concepts such as leg spring stiffness and wind resistance. The STRYD^®^ power meter is a power estimator for running and is currently one of the most widely used devices, worn in the runner’s shoe. According to existing research, it is the most reliable portable power meter on the market [5]. Furthermore, since it requires no calibration, it is highly user-friendly. The STRYD^®^ is a triaxial accelerometer with GPS that integrates elevation and the athlete’s weight, and it also incorporates a gyroscope and a barometer in a compact 9.1-g format. It provides data—some of which are spatiotemporal—that have proven to be valid and reliable [6]. As a high-precision accelerometer, it converts these data into quantifiable metrics for performance analysis [7].

During activity, it measures foot orientation along three axes: horizontal, vertical, and lateral. Unlike the power meters used in cycling, which directly measure applied power output, STRYD^®^ estimates this parameter using a series of formulae. Among the metrics offered by STRYD^®^, those related to biomechanical efficiency and athletic performance stand out. Parameters such as stride length, cadence, ground contact time, and leg stiffness have been associated with variations in running economy, energy expenditure, and fatigue-related responses [8].

The ability to monitor these factors in real time may assist coaches and athletes in making data-informed decisions during training planning and monitoring [9].

Data provided by STRYD^®^ have been widely cited in the scientific literature and used in numerous studies. Power output (PO) is expressed in watts and reflects the intensity of effort during running [10]. It has been proposed as an indicator of running intensity and has shown associations with oxygen consumption (VO2) and energy expenditure under specific testing conditions [11]. Cadence (C) is defined as the frequency of steps per minute during running [12]. Vertical oscillation (VO) reflects the variation in the height of the center of mass during running [13]. Greater vertical oscillation may increase the mechanical work performed against gravity and has been associated with higher energy expenditure in some running conditions [14]. Stride length (SL), defined as the distance covered between the same foot striking the ground twice, has been extensively studied because its interaction with cadence may influence running economy, performance, and injury-related factors [15]. Ground contact time (GCT) refers to the time interval during which the foot remains in contact with the ground while running [16] and has been identified as one of several factors associated with the metabolic cost of running [17]. Leg stiffness (ST) represents the musculoskeletal system’s ability to absorb and reuse elastic energy with each step. It has been associated with running economy, although the strength of this relationship may vary across populations and running conditions [18]. Flight time (FT) is defined as the duration of the aerial phase in the running cycle during which neither foot touches the ground [19]. Estimated VO_2_ refers to the measure of the body’s capacity to capture, transport, and use oxygen during aerobic exercise: in running, it is typically expressed in milliliters of oxygen per kilogram of body mass per minute (mL·kg^−1^·min^−1^) [20]. Finally, form power (FP) represents the power output generated associated with changes in the athlete’s vertical movement [7]. It has also been described as an estimate of the power a runner could have maintained, with the same physiological cost, if their power output had been perfectly constant [1].

Previous reviews have focused primarily on the validity, reliability and technical characteristics of electrical devices and wearable technologies in use, including STRYD^®^. However, as the evidence base has grown, the focus of research has shifted from establishing the accuracy of the device to understanding the practical value of the information it provides.

Therefore, the purpose of this review was not to reassess the validity or reliability of STRYD^®^, but to summarize how this technology has been used in the scientific literature and the insights derived from its application. In particular, we sought to examine whether STRYD^®^ metrics provide meaningful information for monitoring and evaluating running performance.

Furthermore, this review was restricted to trained runners, the population in which power-based training is most commonly implemented and where the potential applications of STRYD^®^ metrics are of greatest practical interest. This focused approach offers a clearer understanding of the current evidence and its relevance to both researchers and practitioners.

## 2. Methods

The systematic review was conducted in accordance with the Preferred Reporting Items for Systematic Reviews and Meta-Analyses (PRISMA) guidelines [21]. A comprehensive literature search was performed between January and February 2025 in the electronic databases PubMed (*n* = 37), Scopus (*n* = 42), and Google Scholar (*n* = 63). The search was restricted to studies published between January 2014 and February 2025, corresponding to the period following the commercial introduction of the STRYD^®^ power meter (Stryd, Boulder, CO, USA).

The search strategy was designed to identify studies investigating the use of the STRYD^®^ power meter and its associated running metrics. Searches were conducted within the title, abstract, and keyword fields whenever permitted by the database. The following Boolean search combinations were applied: (“Running” AND “Stryd”), (“Running” AND “Stryd” AND “Power”), (“Running” AND “Power”), and (“Running” AND (“Stryd” OR “Power”)).

Google Scholar was included as a supplementary source to maximize study identification and reduce the risk of missing relevant studies. Searches were restricted to the same publication period and conducted using the same search terms applied in PubMed and Scopus. Results were ordered according to Google Scholar’s relevance algorithm, and screening was performed until consecutive records no longer met the predefined eligibility criteria. Although exact reproducibility cannot be guaranteed due to the dynamic nature of Google Scholar indexing, the process was standardized through predefined search terms, a fixed publication period, and consistent eligibility assessment procedures.

Given the limited number of publications identified during the preliminary search, no additional restrictions regarding study design, participant characteristics, performance level, or publication type were applied at the search stage in order to maximize search sensitivity and minimize the risk of excluding potentially relevant studies. Searches were limited to studies involving human participants and publications written in English or Spanish.

All retrieved references were imported into Zotero (Corporation for Digital Scholarship, Vienna, VA, USA), where duplicate records were identified and removed prior to screening. In total, 142 records were identified through database searching.

Two reviewers (E.B. and J.M.G.R.) independently screened titles and abstracts for eligibility. Full-text versions were obtained for studies considered potentially relevant and assessed according to predefined inclusion and exclusion criteria. Disagreements were resolved through discussion until consensus was reached, with consultation from a third researcher (C.R.) when necessary.

The inclusion criteria were: (i) studies published between 2014 and 2025; (ii) peer-reviewed articles available in English or Spanish; (iii) research analysing the use of running power meters, specifically STRYD^®^; and (iv) studies including analyses of kinematic, kinetic, and/or spatiotemporal metrics in trained runners. Studies were excluded if they were: (i) duplicate records; (ii) opinion articles, narrative reviews, or technical reports lacking empirical data; or (iii) unavailable in full text.

Data from the included studies were extracted using a purpose-designed data extraction form completed independently by two reviewers. When reported information was unclear or incomplete, Supplementary Materials were examined to clarify the relevant data. No automation tools were used during the screening or data extraction processes and contact with study authors was not required.

**Figure 1:**
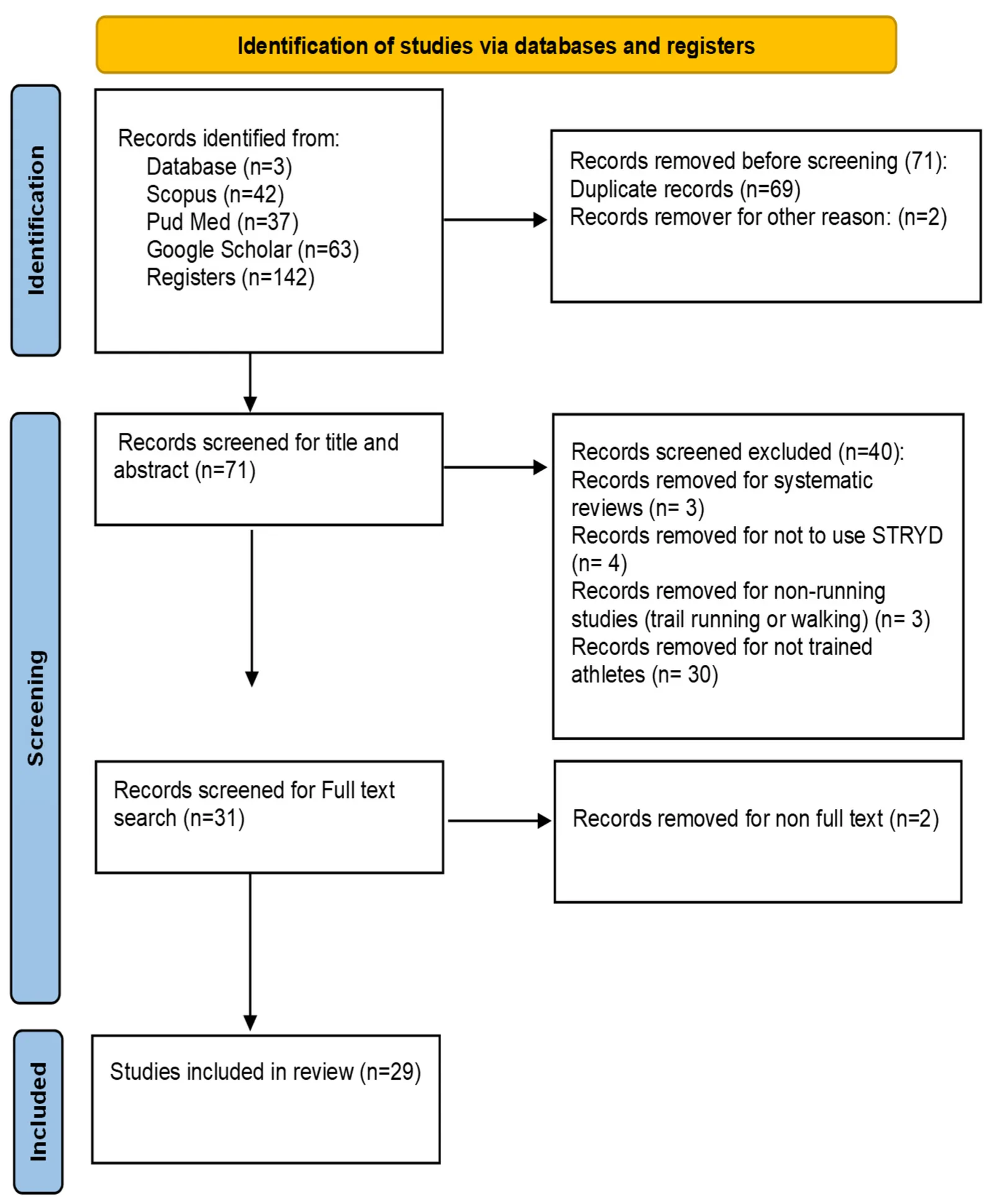
PRISMA flow diagram.

In addition, the reference lists of all included studies were manually screened to identify any potentially eligible articles not retrieved through the electronic database search. Following the selection process, 29 studies met the eligibility criteria and were included in the qualitative synthesis. The study selection process is presented in the PRISMA flow diagram (Figure 1).

The review was retrospectively registered in the Open Science Framework (OSF; https://doi.org/10.17605/OSF.IO/D3N6Y). All eligibility criteria, search procedures, data extraction methods, and risk-of-bias assessments were predefined and conducted in accordance with the PRISMA 2020 guidelines. In addition, the complete search strategy is reported in Supplementary Material S1 to ensure methodological transparency, reproducibility, and verification of the review process.

Data from the selected studies were extracted, taking into account the different parameters used in each study. These parameters, relevant to this review, included power output (PO), ground contact time (GCT), vertical oscillation (VO), cadence (C), flight time (FT), stride length (SL), form power (FP), and stiffness (ST). Information regarding the primary objectives of the included studies was collected to characterise the evidence base and facilitate narrative comparisons between studies.

Studies were allocated to synthesis groups according to their primary research objective and outcome measures. Four thematic domains were identified: (1) physiological validity of running power, (2) validity, reliability and reproducibility of the STRYD device, (3) biomechanical and kinetic responses of running power metrics under different running conditions, and (4) relationships between running power, running economy, and endurance performance.

Owing to the heterogeneity in study designs, participant characteristics, testing protocols, and outcome measures, a quantitative meta-analysis was not conducted. Instead, a narrative synthesis was performed. Data were summarized in evidence tables and synthesized within each thematic domain. Potential sources of heterogeneity were explored qualitatively through comparisons of study design, participant characteristics, testing environment, outcome variables, and methodological approaches. Likewise, a formal assessment of reporting bias was not feasible, as the methodological diversity and lack of comparable quantitative outcomes precluded such analyses. No formal sensitivity analyses were undertaken for the same reasons.

Given the absence of quantitative synthesis and the heterogeneity of the available evidence, a formal GRADE assessment was not performed. Instead, the overall certainty of evidence within each thematic domain was evaluated narratively by considering the number of available studies, their methodological quality, the consistency of findings across studies, and the degree of heterogeneity in study populations, protocols, and outcomes. Based on these criteria, certainty of evidence was classified as high, moderate, low, or very low (Supplementary Material S2).

Comparisons were performed according to the primary study objective, the STRYD^®^ metrics evaluated, and whether a study analysed a single metric or multiple metrics. These factors were used to determine how studies contributed to the narrative synthesis. Study findings were presented narratively and organised in tables according to the objectives and metrics assessed in each study.

The quality of eligible studies was assessed using the risk of bias scale developed by Hindle et al. [22]. This scale is based on other evaluation checklists and has been used previously for the assessment of sports research [23,24]. Sixteen standards were used to evaluate article quality: three on study design, four on sample characteristics, four on methodology, and five on results and discussion. One point was awarded for each standard met, up to a maximum total of 16 points. No half points were awarded. The risk of bias score was subsequently determined based on the total number of points awarded. Articles scoring 11–16 points were categorized as having a low risk of bias. Articles scoring 6–10 points were categorized as having a moderate risk of bias, while studies with a score of 0–5 were categorized as having a high risk of bias.

## 3. Results

### 3.1. Characteristics of the Studies Selected

A total of 142 studies were identified (Figure 1). The reference list of selected manuscripts was also examined for other potentially eligible manuscripts. After the removal of duplicates and elimination of papers based on title and abstract screening, 71 manuscripts remained. The studies that did not match the eligibility criteria were discarded for one or more of the following reasons: (i) systematic review studies; (ii) studies that did not use a STRYD^®^ potentiometer; (iii) studies that did not study running (trail running or walking); and (iv) studies that contained samples of untrained athletes. After this screening process, 29 full-text articles were assessed as eligible for inclusion in this systematic review, as the full text was not found for two articles.

### 3.2. Study Design

In this systematic review, we identified several parameters that contributed to the synthesis of the evidence.

Regarding the temporal orientation of the study, all included studies were cross-sectional (*n* = 29). The included studies described different kinetic and/or spatiotemporal variables, including estimated power output, ground contact time, vertical oscillation, cadence, flight time, stride length, form power, and stiffness (Table 1).

### 3.3. Characteristics of the Participants

A total of 450 athletes (men and women) were included in this systematic review. All were accustomed to undertaking a minimum of three weekly running training sessions. The mean number of participants per study in this review was 15.52 ± 6.56.

### 3.4. Methodological Quality and Risk of Bias

The quality assessment of the included studies was conducted independently by two reviewers (EB and JMGR). Table 2 summarizes the quality and risk of bias of the included studies. The mean score was 12 ± 2 (low risk of bias). No study had a score of 0–5 (high risk of bias), 3 studies had a score between 6 and 10 points (moderate risk of bias) [32,40,41], and 26 studies scored 11–16 points (low risk of bias) [5,6,7,10,25,26,27,28,29,30,31,33,34,35,36,37,38,39,42,43,44,45,46,47,48,49]. The methodological quality assessment was considered during the interpretation of the evidence, with findings from studies presenting a moderate risk of bias interpreted with greater caution and evaluated within the context of the overall body of evidence. Consequently, the practical implications and recommendations proposed in this review are primarily supported by studies exhibiting a low risk of bias, increasing confidence in the validity and applicability of the reported findings. A detailed outline of the assessment criteria is provided in Table 2.

### 3.5. Frequency of Use of Variables

**Figure 2:**
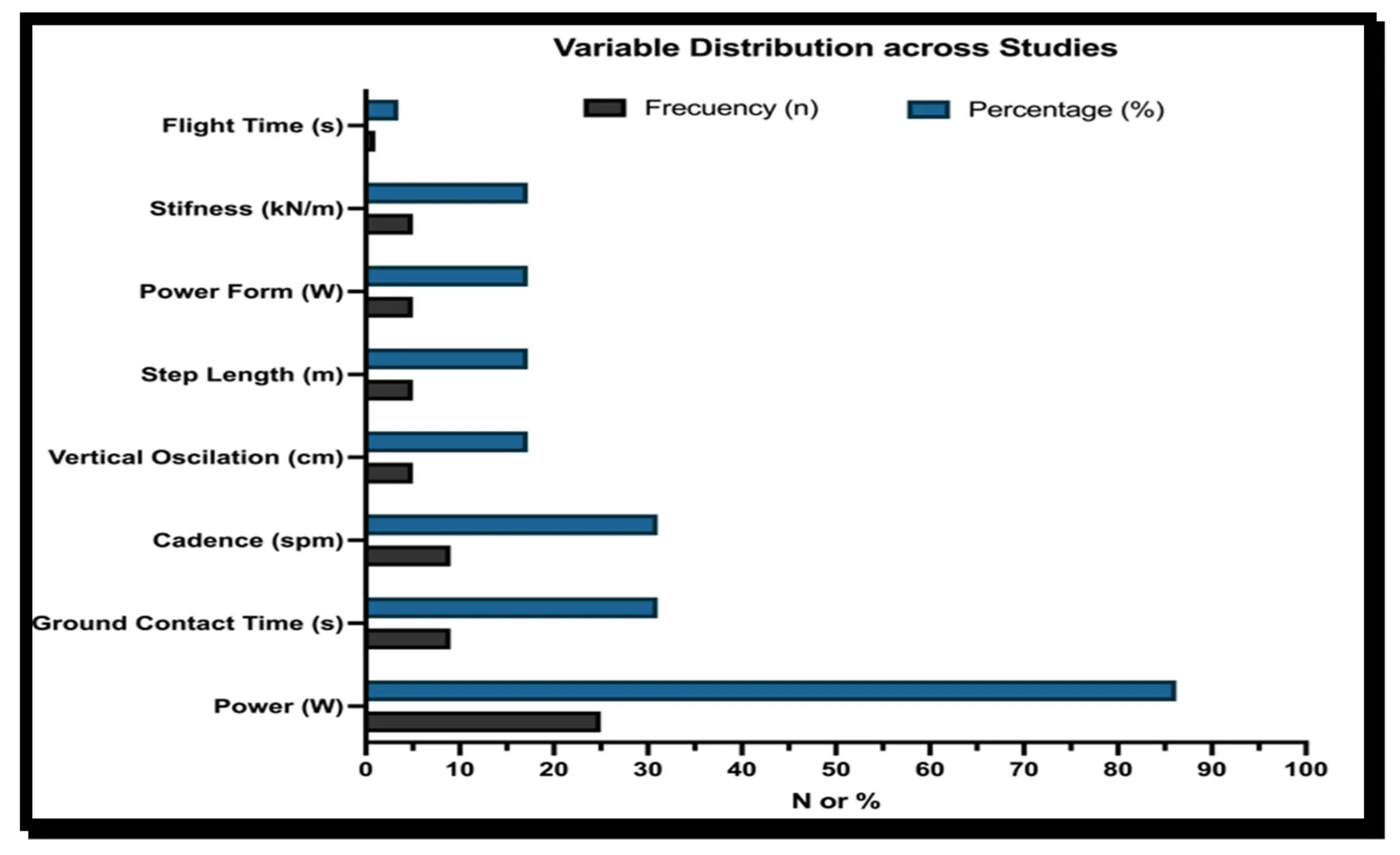
Use and frequency of variables.

The variables measured in the various studies provided an indication of how the STRYD^®^ power meter measured in the various studies provided an indication of how the STRYD^®^ power meter is used in the analysis of running performance. In the 29 studies, power output (PO) was, unsurprisingly, the most frequently used variable. It was investigated in 25 of the 29 studies (86.21%) [5,7,10,25,26,27,28,29,30,31,32,33,35,36,37,38,40,41,42,43,44,45,46,48,49]. Next was ground contact time (GCT) [6,10,34,35,36,39,47,48,49] and cadence (C) [5,6,7,10,34,36,39,48,49] with 9 instances (31.03%). Vertical oscillation (VO) [10,34,47,48,49], stride length (SL) [6,34,36,47,49], form power (FP) [5,7,28,48,49] and stiffness (ST) [5,34,47,48,49] were all investigated in 5 studies (17.24%) and, flight time (FT) [6] was investigated on 1 occasion (3.45%) (Figure 2).

When the studies were analyzed according to their primary research objective, distinct patterns in the use of STRYD^®^ metrics emerged. In the studies focused on the physiological validity of running power, PO was the predominant variable and was included in all studies, whereas biomechanical metrics were only occasionally assessed. Similarly, studies investigating the validity, reliability, and reproducibility of the STRYD^®^ device primarily focused on PO, with only limited evaluation of additional variables such as C, GCT, FT, SL, FP and ST. Studies examining biomechanical and kinetic responses under different running conditions showed a more balanced distribution of variables. Alongside PO, variables such as GCT, C, VO, SL, ST and FP were frequently investigated, reflecting a broader interest in the biomechanical information provided by the device. Studies relating STRYD^®^ metrics to running economy and performance were again dominated by PO, which was assessed in all studies within this category. However, GCT, C and FP were also commonly included, suggesting that these variables are considered potentially relevant when evaluating performance-related outcomes. By contrast, FT was rarely investigated across all research areas and appeared in only one study.

Overall, these findings indicate that the selection of STRYD^®^ metrics is strongly influenced by the specific objective of the research, with physiological and performance studies focusing predominantly on PO, whereas biomechanical investigations tend to incorporate a wider range of kinematic and kinetic variables.

### 3.6. Variables per Study

**Figure 3:**
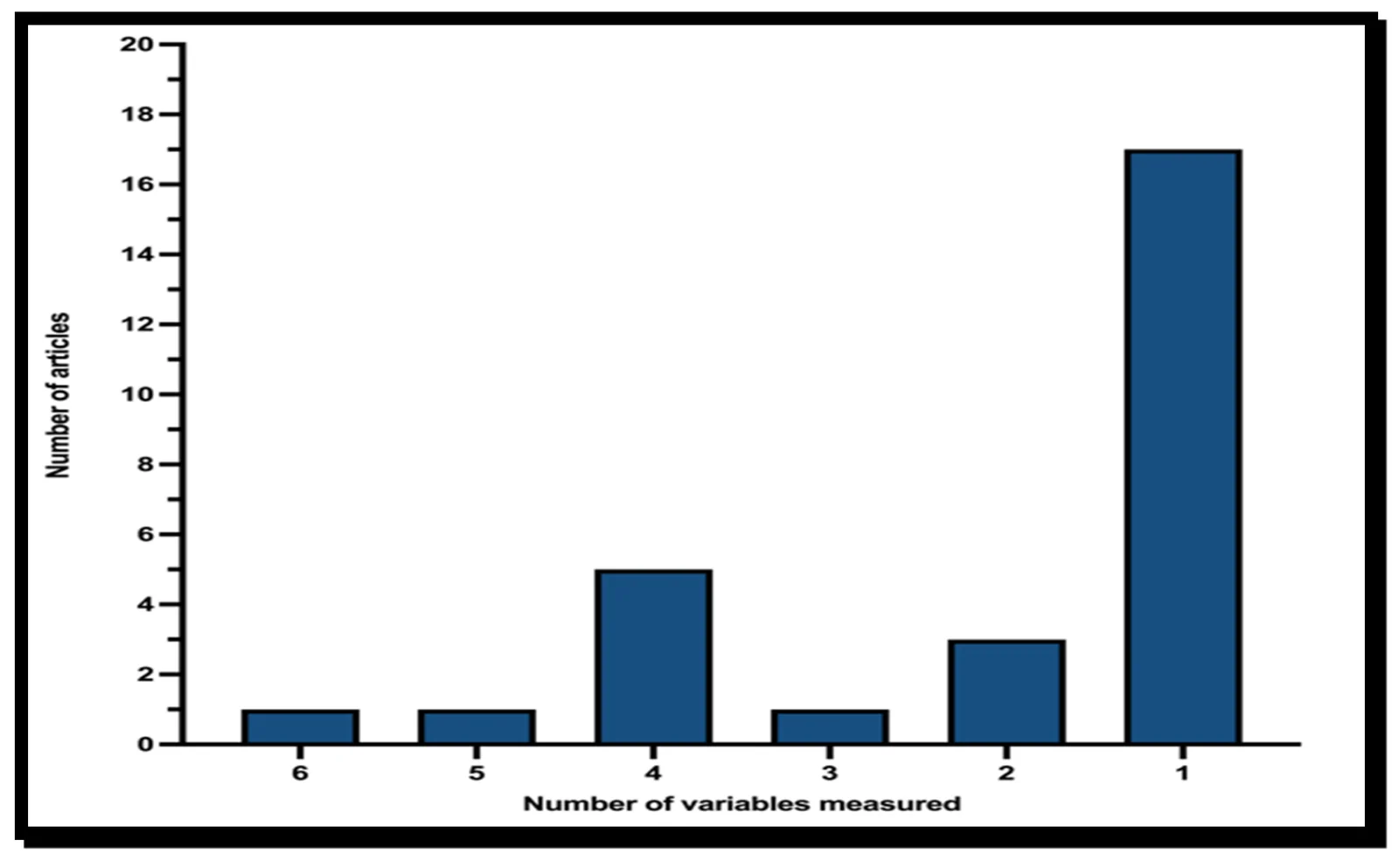
Distribution of articles by number of parameters analyzed.

The review included articles that varied considerably in the number of variables used (Figure 3). Thus, the most variables considered in an article was 7 [49]. One study examined 6 variables [48], one looked at 5 [34], 5 articles examined 4 variables [5,6,10,36,47], 1 article investigated 3 [7], 3 articles studied 2 [28,35,39] and 17 articles assessed a single variable, power output [25,26,27,29,30,31,32,33,37,38,40,41,42,43,44,45,46].

### 3.7. Relationship Between Measured Variables and Study Objectives

Table 3 shows the number of times per study that each variable was involved in the primary study objective. Unsurprisingly, PO was the most frequently studied variable, appearing 16 times [5,7,10,26,28,29,30,32,36,38,40,41,42,44,46,49], followed by critical power (CP) with 10 occurrences [25,27,31,33,35,37,42,43,44,46], GCT on 6 occasions [6,34,39,47,48,49], C on 5 occasions [6,28,34,39,48], and 4 occasions each for FP [7,28,48,49], ST [34,47,48,49] and VO [34,47,48,49]. SL appeared on 3 occasions [6,47,48]. The following variables appeared on 2 occasions: VO_2_ [5,10], speed (S) [26,49], running economy (RE) [36,39] and running effectiveness (REF) [48,49], and the remaining variables occurred once: critical speed (CS) [27], FT [6], maximum running speed (MRS) [49], horizontal power (HP) [49], vertical ratio (VR) [49] and maximal oxygen consumption (VO_2_max) [49].

### 3.8. Certainty of Evidence Across Thematic Domains

The certainty of the evidence varied across thematic domains. The evidence supporting the validity, reliability, and reproducibility of the STRYD device was considered high due to the consistency of the findings and the methodological robustness of the available studies. The physiological validity of running power and biomechanical responses derived from STRYD metrics was supported by evidence of moderate certainty due to heterogeneity among protocols and results, despite generally consistent findings. In contrast, the evidence linking STRYD-derived metrics to running economy and endurance performance was considered to be of low certainty due to the limited number of studies, small sample sizes, and variability in the reported associations.

## 4. Discussion

The STRYD^®^ power meter is a relatively new technology that provides physiological and biomechanical information that may be useful for training monitoring and performance assessment in runners. Jaen-Carrillo et al. [50] examined the repeatability of various devices designed to measure power output (STRYD^®^, Runscribe^®^, Garmin Running Power^®^ and Polar Vantage V^®^), and their concurrent validity with respect to VO_2_. The findings showed that STRYD^®^ was the device with the highest repeatability in power output estimation. Another study by García-Pinillos et al. [51] demonstrated that the reliability of STRYD^®^ in estimating power output at a constant speed was consistent across both short and long intervals.

The results show that of the variables obtained by STRYD^®^, the one most frequently used in the scientific literature has been PO, which also aligns with the primary objective of these studies, appearing in 86.21% of the studies analyzed. This finding may be based on the fact that PO has a long history as a metric indicative of important parameters for running. Van Rassel et al. [52] found that PO was strongly correlated with VO_2_ and maximum lactate steady state (MLSS) speed, concluding that it was a good indicator of differences in intensity and useful as an estimator of mechanical efficiency. Another study indicated that power output measured using the STRYD^®^ power meter yielded estimates with lower error than speed for certain endurance performance models [53]. Thus, power output may represent a useful parameter for training monitoring and prescription, although further intervention studies are required to determine its direct impact on training outcomes, as it is strongly related to performance and is a very straightforward variable to interpret.

C was the second most frequently used parameter in the studies included in this review, and, like GCT, it was found on 10 occasions. These two parameters were found to be reliable values obtained by STRYD^®^ at varying speeds. The same applied to VO_2_ and FP [54]. Thus, the relationship between PO and C and GCT can be interpreted thanks to their measurement by STRYD^®^ under controlled conditions. They also enable the quantification of mechanical cost and stride rate, which are factors closely linked to running performance and economy.

Other recent studies have linked ST to running performance. A higher ST is associated with better performance in elite runners, as it allows for greater reuse of elastic energy during the flight phase [55]. A systematic review also found that a higher VO_2_ and ST are associated with better RE, implying superior performance [14].

CP was also the second most frequently studied parameter in this systematic review. This parameter can be provided by the STRYD^®^ power meter via its website, and is relevant to endurance exercise. CP represents the boundary between steady-state and non-steady-state exercise [56]. An article by Olaya-Cuartero et al. [31] strongly linked CP in the half-marathon with the CP obtained using a test. Van Rassel et al. [27] found that CP occurred at an intensity similar to critical speed (CS), with the former aligning with actual performance and serving as a reference for training prescription. CP also occurs at an intensity very close to the lactate threshold, thus offering a means of defining training zones [25].

With regard to the number of variables that studies included in their analyses, the results showed considerable variation: only a small group of studies (3) analyzed more than four variables, indicating a lack of standardization in the use of power meters in research, which limits robust comparison between studies. Those that analyzed the most variables estimated by STRYD^®^ (up to seven [49] and six [48] variables) had among their main research objectives the analysis of biomechanical variables for running performance. Studying and understanding the biomechanical parameters provided brings us closer to identifying the relationship between them and how they interact. This is one of the main findings of this review, which positions the STRYD^®^ power meter as a technology used in the pursuit of knowledge to improve running performance. Thus, a study by Jaén-Carrillo et al. [48] concluded that the biomechanical factors of GCT and FP are good indicators of running economy and performance. Other study by Pardo-Albiach et al. [49] found that faster individuals had higher power-to-weight ratios and SL, and lower GCT and FP ratios. This indicates that, to run faster, athletes must also manage their power output correctly.

An additional finding of this review was the distinct distribution of STRYD^®^ metrics according to the primary objective of the studies. PO was the predominant variable in research examining physiological validity, device reliability, and performance-related outcomes, highlighting its central role in the scientific application of running power. In contrast, studies focused on biomechanical responses incorporated a broader range of variables, including GCT, C, VO, SL, ST and FP. This pattern suggests that, while PO remains the principal metric used to investigate training intensity and performance, the biomechanical metrics provided by STRYD^®^ are increasingly being used to explore the mechanisms underlying running performance and movement adaptations.

The differences observed between research areas also reflect the evolution of the scientific interest in STRYD^®^ technology. Early investigations were primarily concerned with validating the accuracy and reliability of power-related measurements, whereas more recent studies have expanded their focus towards understanding the physiological, biomechanical, and performance-related information derived from these metrics. This transition supports the view that the scientific relevance of STRYD^®^ extends beyond the estimation of running power alone and increasingly involves the interpretation of multiple variables to better understand running performance.

For all these reasons, the STRYD^®^ power meter is a reliable technology, providing metrics that may assist training monitoring, performance assessment and the evaluation of running characteristics and identifying potential improvements for runners and their coaches.

However, the STRYD^®^ power meter has some limitations. The first is that STRYD^®^ is a technology that does not measure directly, but rather provides estimates of power output, without disclosing the algorithms used in its software. Although numerous studies have reported acceptable validity and reliability for metrics derived from STRYD^®^, the lack of transparency regarding the underlying calculations limits the ability to independently verify how these estimates are generated. This also makes it difficult to directly compare them with alternative approaches for estimating running power and may influence the interpretation of findings across devices, studies, and future software updates. Consequently, the available evidence should be interpreted primarily in terms of the reported validity, reliability, and practical associations for STRYD^®^ metrics, rather than assuming direct equivalence with externally measurable mechanical power. Publication bias and selective outcome reporting cannot be ruled out. Studies reporting statistically significant or favorable results regarding STRYD-derived metrics may have been more likely to be published than studies reporting null or negative findings, which may have influenced the overall conclusions of this review. Another limitation identified in this review is the use of different population groups in relation to their performance, which may lead to variations in the metrics provided. Finally, environmental factors (wind, temperature, humidity, surface type and footwear) may need to be controlled for in future studies to standardize results, as these were not always taken into account in the reviewed studies and, according to Cerezuela-Espejo et al. [45], could alter the results.

Importantly, no studies were identified as presenting a high risk of bias. Therefore, the conclusions of this review are based on evidence derived predominantly from studies of low to moderate methodological risk. Nevertheless, the methodological quality ratings were considered when interpreting the findings, giving greater weight to conclusions supported by studies with lower risk of bias and higher methodological rigor.

Current evidence enables coaches and runners to use new technologies for measuring and managing training load. According to Aubry et al. [10], PO can be a good indicator of running intensity. Van Rassel et al. [52] indicated that PO obtained via STRYD^®^ is particularly useful as an intensity control tool, and another study by Olaya-Cuartero et al. [37] suggested that CP estimated using STRYD^®^ can be a good predictor of performance, making it relevant for load management and planning. Providing kinematic data on running may assist the assessment of factors associated with performance and injury-related risk.

Sensors such as STRYD^®^ can be useful for measuring biomechanical parameters such as C, SL, ST, GCT and VO, which can help to improve performance in endurance athletes [57]. A study by Short et al. [58] reported that biomechanical parameters such as VO, FP, ST and GCT provided by the STRYD^®^ power meter explained 90% of the variance in critical speed. This allows coaches and athletes to refine kinematic aspects during a run and, more importantly, to know which characteristics to focus on. Another study by Van Hooren et al. [14] concluded that running biomechanics could account for between 4% and 12% of the variation in ER between individuals when considered in isolation, and this proportion may increase when different variables are combined. Austin et al. [7] suggested in one of their studies that there is a positive relationship between PO and FP and RE expressed in terms of oxygen cost and unit caloric cost. Finally, a study by Aubry et al. [10] on the individual mechanical components of power indicated that improved efficiency was associated with a decrease in GCT (r = 0.56), VO (r = 0.46) and C (r = 0.37) on the treadmill for a recreational group. Thus, in this recreational population, this training tool could be useful for providing feedback on various running dynamics known to influence running economy. The available evidence was also characterized by substantial heterogeneity in study populations, testing protocols, and outcome measures. These differences may partly explain the variability in reported findings and limited the possibility of quantitative comparison across studies.

Although the available evidence supports the validity, reliability and physiological or biomechanical relevance of various STRYD^®^ metrics, most of the included studies were observational or validation-based in nature. Therefore, caution should be exercised when extrapolating these findings to directly improve outcomes in training, sporting performance or injury prevention. Further longitudinal and interventional studies are needed to determine whether the systematic use of metrics derived from STRYD^®^ leads to significant improvements in these applied outcomes.

## 5. Conclusions

This systematic review summarises the current evidence on the use of the STRYD^®^ power meter in trained runners. The available literature supports STRYD^®^ as a valid and reliable technology for estimating running power and various biomechanical variables under different test conditions. Power output was the most frequently investigated metric and was the variable most consistently examined in relation to physiological indicators such as VO_2_, maximum lactate steady-state speed, and running economy. Cadence and ground contact time were also frequently examined, whilst vertical oscillation, stiffness, stride length and form power received relatively less attention. Time of flight was rarely investigated.

The available literature suggests that the scientific use of STRYD^®^ has evolved from the validation of power measurements towards the exploration of the physiological, biomechanical and performance-related information provided by its metrics. However, the current body of evidence is based primarily on observational, validation and reliability studies. Therefore, STRYD^®^ metrics should be regarded as potentially useful tools for monitoring and assessing training and performance, rather than as direct evidence of an improvement in outcomes. Further longitudinal and interventional studies are needed to determine whether the systematic use of these metrics leads to significant improvements in training prescription, performance and other applied outcomes.

## Figures and Tables

**Table 1:** List of selected studies and variables obtained by STRYD^®^.

Authors	Year	No. of Subjects	PO	GCT	VO	C	FT	SL	FP	ST	Total
Aubry et al. [10]	2018	24	x	x	x	x					4
Ruiz-Alias et al. [25]	2022	15	x								1
Van Rassel et al. [26]	2023	15	x								1
Van Rassel et al. [27]	2023	10	x								1
García-Pinillos et al. [6]	2021	18		x		x	x	x			4
Cartón-Llorente et al. [28]	2022	31	x						x		2
Cerezuela-Espejo et al. [5]	2021	12	x			x			x	x	4
Ruiz-Alias et al. [29]	2023	15	x								1
Ruiz-Alias et al. [30]	2024	15	x								1
Olaya-Cuartero et al. [31]	2024	24	x								1
Coppi et al. [32]	2018	8	x								1
Olaya-Cuartero et al. [33]	2023	18	x								1
Olaya-Cuartero et al. [34]	2021	4		x	x	x		x		x	5
Dearing et al. [35]	2023	20	x	x							2
Baumgartner et al. [36]	2021	32	x	x		x		x			4
Olaya-Cuartero et al. [37]	2023	9	x								1
García-Pinillos et al. [38]	2019	18	x								1
Rodrigo-Carranza et al. [39]	2024	21		x		x					2
Shearer et al. [40]	2018	8	x								1
Lara et al. [41]	2018	8	x								1
Ruiz-Alias et al. [42]	2024	15	x								1
Ruiz-Alias et al. [43]	2023	15	x								1
Ruiz-Alias et al. [44]	2024	15	x								1
Cerezuela-Espejo et al. [45]	2020	10	x								1
Ruiz-Alias et al. [46]	2024	15	x								1
Olaya-Cuartero et al. [47]	2024	8		x	x			x		x	4
Jaén-Carrillo et al. [48]	2026	15	x	x	x	x			x	x	6
Austin et al. [7]	2018	17	x			x			x		3
Pardo Albiach et al. [49]	2021	15	x	x	x	x		x	x	x	7
Total			25	9	5	9	1	5	5	5	

Note: Color coding: White = physiological validity of running power; light blue = validity, reliability, and reproducibility of the Stryd device; medium blue = biomechanical and kinetic responses under different running conditions; dark blue = associations with running economy and performance. Abbreviations: PO, power output; GCT, ground contact time; VO, vertical oscillation, C, cadence; FT, flight time; SL, stride length; FP, form power; ST, stiffness.

**Table 2:** Quality assessment of running and power articles in trained individuals.

Study	Publication Year	1	2	3	4	5	6	7	8	9	10	11	12	13	14	15	16	Total
Olaya-Cuartero et al. [31]	2024	x	x	x	x	x	x		x	x	x	x	x	x	x	x	x	15
Olaya-Cuartero et al. [33]	2023	x	x	x	x	x			x	x	x	x	x	x	x	x	x	14
Dearing et al. [35]	2023	x	x	x	x	x			x	x	x	x	x	x	x	x	x	14
Baumgartner et al. [36]	2021	x	x	x	x	x			x	x	x	x	x	x	x	x	x	14
García-Pinillos et al. [38]	2019	x	x	x	x	x			x	x	x	x	x	x	x	x	x	14
Rodrigo-Carranza et al. [39]	2024	x	x	x	x	x			x	x	x	x	x	x	x	x	x	14
Ruiz-Alias et al. [44]	2024	x	x	x	x	x			x	x	x	x	x	x	x	x	x	14
Ruiz-Alias et al. [42]	2024	x	x	x	x	x			x		x	x	x	x	x	x	x	14
Olaya-Cuartero et al. [47]	2024	x	x	x	x	x			x	x	x	x	x	x	x	x	x	14
Jaén-Carrillo et al. [48]	2026	x	x	x	x	x			x	x	x	x	x	x	x	x	x	14
Van Rassel et al. [26]	2023	x	x	x		x			x	x	x	x	x	x	x	x	x	13
García-Pinillos et al. [6]	2021	x	x	x	x	x			x	x	x	x	x	x	x		x	13
Ruiz-Alias et al. [29]	2023	x	x	x	x	x			x	x	x	x	x	x	x	x		13
Olaya-Cuartero et al. [34]	2021	x	x	x		x		x	x	x	x	x	x	x	x	x		13
Ruiz-Alias et al. [30]	2024	x	x	x	x	x			x	x	x	x	x	x	x	x		13
Austin et al. [7]	2018	x	x	x	x				x	x	x	x	x	x	x	x	x	13
Pardo Albiach et al. [49]	2021	x	x		x	x			x	x	x	x	x	x	x	x	x	13
Aubry et al. [10]	2018	x	x	x		x			x	x	x	x	x	x	x		x	12
Ruiz-Alias et al. [25]	2022	x	x		x	x			x	x	x	x	x	x	x		x	12
Van Rassel et al. [27]	2023	x	x	x		x			x	x	x	x	x	x	x		x	12
Cartón-Llorente et al. [28]	2022		x	x	x	x			x		x	x	x	x	x	x	x	12
Cerezuela-Espejo et al. [5]	2021	x	x	x		x			x		x	x	x	x	x	x	x	12
Olaya-Cuartero et al. [37]	2023	x	x	x	x	x			x	x	x	x	x	x			x	12
Ruiz-Alias et al. [43]	2023	x	x		x	x			x	x	x	x	x	x	x	x		12
Cerezuela-Espejo et al. [45]	2020	x	x	x	x	x			x		x	x	x	x	x		x	12
Ruiz-Alias et al. [46]	2024	x	x			x			x	x	x	x	x	x	x		x	11
Coppi et al. [32]	2018	x	x	x		x				x	x	x						7
Shearer et al. [40]	2018	x	x	x		x			x	x						x		7
Lara et al. [41]	2018	x	x	x		x			x	x								6

Note: Color coding: White = physiological validity of running power; light blue = validity, reliability, and reproducibility of the Stryd device; medium blue = biomechanical and kinetic responses under different running conditions; dark blue = associations with running economy and performance. The items had the following characteristics: (1) study design was stated clearly; (2) the study objective/purpose was clearly stated; (3) the study had a clearly testable hypothesis; (4) the study clearly stated the inclusion criteria for participants; (5) the characteristics of the population were well detailed; (6) the study population was representative of the intended population at whom the research was aimed; (7) a justification for the selection of the sample/study population size was provided; (8) the methods used throughout testing were well detailed; (9) the measurement tools used throughout the study were reliable and had been validated; (10) detail on the statistical methods used was provided; (11) the statistical methods used to analyze the data were appropriate; (12) the results of the study were well detailed; (13) the information provided in the paper was sufficient to allow the reader to make an unbiased assessment of the study findings; (14) confounding factors within the study were identified; (15) study funding/conflicts of interest were acknowledged; and (16) limitations to the study were identified. Low risk of bias (11–16), Satisfactory risk of bias (6–10), High risk of bias (0–5).

**Table 3:** Key variables for the primary outcome by study.

Authors	Year	Key Variables for the Primary Outcome Measure
Aubry et al. [10]	2018	PO and VO_2_
Ruiz-Alias et al. [25]	2022	CP
Van Rassel et al. [26]	2023	PO and S in MMSS
Van Rassel et al. [27]	2023	CP CS
García-Pinillos et al. [6]	2021	GCT, FT, SL, and C
Cartón-Llorente et al. [28]	2022	PO, C, FP
Cerezuela-Espejo et al. [5]	2021	PO and VO_2_
Ruiz-Alias et al. [29]	2023	PO
Ruiz-Alias et al. [30]	2024	PO
Olaya-Cuartero et al. [31]	2024	CP
Coppi et al. [32]	2018	PO
Olaya-Cuartero et al. [33]	2023	CP
Olaya-Cuartero et al. [34]	2021	ST, GCT, VO_2_ and C
Dearing et al. [35]	2023	CP
Baumgartner et al. [36]	2021	RE, PO
Olaya-Cuartero et al. [37]	2023	CP
García-Pinillos et al. [38]	2019	PO
Rodrigo-Carranza et al. [39]	2024	RE, C and GCT
Shearer et al. [40]	2018	PO
Lara et al. [41]	2018	PO
Ruiz-Alias et al. [42]	2024	CP and W(PO)
Ruiz-Alias et al. [43]	2023	CP
Ruiz-Alias et al. [44]	2024	CP and W(PO)
Cerezuela-Espejo et al. [45]	2020	PO
Ruiz-Alias et al. [46]	2024	CP
Olaya-Cuartero et al. [47]	2024	GCT, ST, VO, and SL
Jaén-Carrillo et al. [48]	2026	GCT, ST, VO, FP, and REF
Austin et al. [7]	2018	PO FP
Pardo Albiach et al. [49]	2021	MRS, PO, ST, VO, HP, S, C, GCT, VR, SL, REF, FP, and VO_2_max

Note: Color coding: White = physiological validity of running power; light blue = validity, reliability, and reproducibility of the Stryd device; medium blue = biomechanical and kinetic responses under different running conditions; dark blue = associations with running economy and performance. Abbreviations: PO (Power output), VO_2_ (oxygen consumption), CP (Critical Power), S (Speed), MMSS (Maximal Metabolic Steady State), CS (Critical Speed), FT (Flight Time), SL (stride length), C (Cadence), FP (Form power), ST (stiffness), VO (vertical oscillation), RE (Running economy), REF (running effectiveness), W (Work), MRS (Maximum Running Speed)., HP (horizontal power), VR (vertical ratio) and VO_2_ max (maximum oxygen consumption).

## Data Availability

The review protocol, complete search strategy, risk-of-bias assessments, and certainty-of-evidence assessments are publicly available through the Open Science Framework (OSF) repository: https://doi.org/10.17605/OSF.IO/D3N6Y.

## Supplementary Materials

The following supporting information can be downloaded at: https://www.mdpi.com/article/10.3390/sports14080364/s1. Supplementary Material S1: Detailed Search Strategy. Supplementary Material S2: Narrative Assessment of the Certainty of Evidence Across Thematic Domains.
