# Supplementary material for: Use of the STRYD® Power Meter and Its Metrics in Trained Runners: A Systematic Review
Source: Sports (Basel). 2026 Aug 21;14(8):364. doi: 10.3390/sports14080364 (PMC13517860; doi:10.3390/sports14080364)
Supplement: Supplementary file 1 [file sports-14-00364-s001.zip › Supplementary Material S1.pdf]

Supplementary Material S1. Detailed Search Strategy

Databases searched

- PubMed
- Scopus
- Google Scholar

Search period

Searches were conducted between January and February 2025. Only studies published between January 2014 and February 2025 were considered.

Search restrictions

- Human participants only.

- English and Spanish language publications.

- No restrictions regarding study design, participant characteristics, performance level, or publication type due to the limited number of publications available on this topic.

PubMed

- Date of search: February 2025
- Search strategy:

Preliminary searches demonstrated that the term "Stryd" captured the vast majority of the literature related to running power assessment, while broader terms substantially increased the number of irrelevant results without identifying additional eligible studies.

("running" AND "Stryd") OR ("running" AND "Stryd" AND "power") OR ("running" AND "power") OR ("running" AND ("Stryd" OR "power"))

- Filters applied: Publication years (2014-2025); Humans; English and Spanish.
- Records retrieved: 37

Scopus

- Date of search: February 2025.
- Search strategy:

Preliminary searches demonstrated that the term "Stryd" captured the vast majority of the literature related to running power assessment, while broader terms substantially increased the number of irrelevant results without identifying additional eligible studies

Search strategy:

TITLE-ABS-KEY ("running" AND "Stryd") OR TITLE-ABS-KEY ("running" AND "Stryd" AND "power") OR TITLE-ABS-KEY ("running" AND "power") OR TITLE-ABS-KEY ("running" AND ("Stryd" OR "power"))

- Filters applied: Publication years (2014-2025); Humans; English and Spanish.
- Records retrieved: 42

<u>Google Scholar</u>

Given the emerging nature of the topic and the relatively small number of available publications, Google Scholar was included as a supplementary search source to maximize study identification and reduce the risk of missing relevant articles.

- Date of search: February 2025
- Search strategy:

"running" AND "Stryd"; "running" AND "Stryd" AND "power"; "running" AND "power"; "running" AND ("Stryd" OR "power"); "Stryd" OR "running" AND "power meter".

- Limited to Academic Journals only
- Filters applied: Publication years (2014-2025); Humans; English and Spanish.
- Records retrieved: 63

Additional search procedures

Study selection and data extraction were performed independently by two reviewers (E.B. and J.M.G.R.). Any disagreements were resolved through discussion until consensus was reached, with consultation of a third reviewer (C.R.) when necessary. Studies considered potentially relevant were retrieved in full text and assessed according to the predefined inclusion and exclusion criteria. Reference lists of all included studies were manually screened to identify potentially relevant articles not captured through the electronic database searches. All references were exported to Zotero, where duplicate records were identified and removed prior to screening.
