# Supplementary material for: Use of the STRYD® Power Meter and Its Metrics in Trained Runners: A Systematic Review
Source: Sports (Basel). 2026 Aug 21;14(8):364. doi: 10.3390/sports14080364 (PMC13517860; doi:10.3390/sports14080364)
Supplement: Supplementary file 1 [file sports-14-00364-s001.zip › Supplementary Material S2.pdf]

Supplementary Material S2. Narrative Assessment of the Certainty of Evidence Across Thematic Domains

Thematic domain	No. of studies	Methodological quality	Consistency of findings	Heterogeneity	Certainty of evidence	Justification
**Physiological validity of running power**	10	Moderate to high. Most studies used controlled experimental protocols and objective physiological measurements (VO_2, ventilatory thresholds, MLSS, CP).	Moderate. Several studies support associations between running power and physiological thresholds, although findings regarding the relationship between running power and metabolic cost/running economy are not entirely consistent.	Moderate. Differences in populations, testing environments (treadmill vs. track), physiological outcomes, and analytical approaches.	**MODERATE**	A relatively large body of evidence supports the physiological relevance of running power, but inconsistencies regarding its ability to reflect metabolic demand prevent a high-certainty rating.
**Validity, reliability and reproducibility of the STRYD device**	6	High. Studies specifically designed to assess reliability, validity, reproducibility, inter-unit and inter-limb agreement.	High. Consistently low measurement error, high ICC values, and similar findings across studies.	Low. Methodologies and evaluated outcomes were comparable across investigations.	**HIGH**	Strong agreement among studies demonstrates that STRYD provides reliable and reproducible measurements under a variety of running conditions.
**Biomechanical and kinetic responses under different running conditions**	7	Moderate. Most studies used experimental or observational designs with objective biomechanical measurements.	Moderate. Several biomechanical metrics showed stable and interpretable responses, although findings varied depending on running conditions and populations.	Moderate to high. Considerable variability in protocols, fatigue conditions, footwear interventions, race settings, and outcome variables.	**MODERATE**	Evidence consistently supports the usefulness of STRYD-derived biomechanical metrics, although substantial methodological heterogeneity limits confidence in some conclusions.
**Relationship with running economy and endurance performance**	6	Moderate. Studies generally employed valid physiological and performance assessments but often included small samples.	Low to moderate. Associations between STRYD metrics and running economy or performance were observed, but their magnitude and relevance varied between studies.	High. Outcomes, participant characteristics, and performance indicators differed substantially across studies.	**LOW**	Although promising relationships have been reported, the limited number of studies, small sample sizes, and inconsistent findings reduce confidence in the overall evidence.
